# Proadrenomedullin N-Terminal 20 Peptides (PAMPs) Are Agonists of the Chemokine Scavenger Receptor ACKR3/CXCR7

**DOI:** 10.1021/acsptsci.1c00006

**Published:** 2021-03-22

**Authors:** Max Meyrath, Christie B. Palmer, Nathan Reynders, Alain Vanderplasschen, Markus Ollert, Michel Bouvier, Martyna Szpakowska, Andy Chevigné

**Affiliations:** † Department of Infection and Immunity, Luxembourg Institute of Health (LIH), Esch-sur-Alzette L-4354, Luxembourg; ‡ Faculty of Science, Technology and Medicine, University of Luxembourg, Esch-sur-Alzette 4365, Luxembourg; § Immunology-Vaccinology, FARAH, Faculty of Veterinary Medicine, University of Liège, Liège BE 4000, Belgium; ∥ Department of Dermatology and Allergy Center, Odense Research Center for Anaphylaxis, University of Southern Denmark, Odense 5230, Denmark; ⊥ Department of Biochemistry and Molecular Medicine, Institute for Research in Immunology and Cancer (IRIC), Université de Montréal, Montreal, H3C 3J7 Quebec, Canada

**Keywords:** ACKR3, CXCR7, PAMP-12, adrenomedullin, MRGPRX2, RAMP

## Abstract

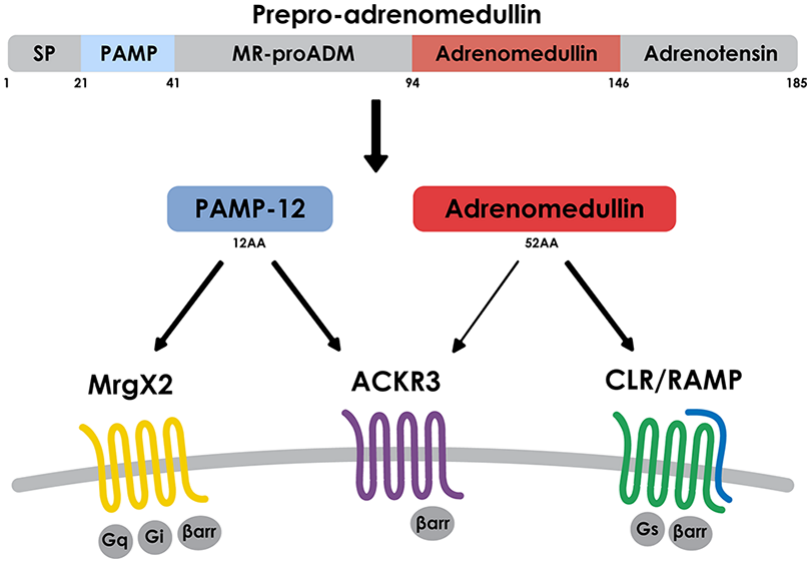

Adrenomedullin (ADM) and proadrenomedullin N-terminal 20 peptide (PAMP) are two peptides with vasodilative, bronchodilative, and angiogenic properties, originating from a common precursor, proADM. Previous studies proposed that the atypical chemokine receptor ACKR3 might act as a low-affinity scavenger for ADM, regulating its availability for its cognate receptor calcitonin receptor-like receptor (CLR) in complex with a receptor activity modifying protein (RAMP). In this study, we compared the activation of ACKR3 by ADM and PAMP, as well as other related members of the calcitonin gene-related peptide (CGRP) family. Irrespective of the presence of RAMPs, ADM was the only member of the CGRP family to show moderate activity toward ACKR3. Remarkably, PAMP, and especially further processed PAMP-12, had a stronger potency toward ACKR3 than ADM. Importantly, PAMP-12 induced β-arrestin recruitment and was efficiently internalized by ACKR3 without inducing G protein or ERK signaling *in vitro*. Our results further extend the panel of endogenous ACKR3 ligands and broaden ACKR3 functions to a regulator of PAMP-12 availability for its primary receptor Mas-related G-protein-coupled receptor member X2 (MrgX2).

Atypical chemokine receptors (ACKRs) are vital regulators of the spatiotemporal distribution of chemokines. ACKRs mediate chemokine internalization, degradation, sequestration, or transcytosis without inducing classical G-protein-mediated signaling.^1^ ACKR3, formerly named CXCR7, is expressed ubiquitously but is most abundantly present in different brain regions, adrenal glands, lymphatic and blood vasculature, heart, and various subsets of immune cells.^2,3^ ACKR3 is a selective scavenger for two endogenous chemokines, CXCL12 and CXCL11, which are also the ligands of CXCR4 and CXCR3, respectively, and for the human herpesvirus 8 (HHV-8)-encoded chemokine vCCL2, as well as the pseudochemokine MIF.^4−6^ Recently, it has also been shown that ACKR3 is a high-affinity scavenger for a broad spectrum of opioid peptides and modulates their availability for classical opioid receptors.^7,8^

ACKR3 regulates embryogenesis, hematopoiesis, neuronal migration, angiogenesis, and cardiac development.^4,7,9^ Genetic knockout of *Ackr3* in mice is associated with cardiomyocyte hyperplasia and disrupted lymphangiogenesis, usually leading to perinatal death due to cardiac valve and ventricular septal defects.^10,11^ However, these defects do not correlate with the CXCL12-CXCR4 signaling axis, suggesting that ACKR3 interaction with ligands other than CXCL12 may be responsible for this phenotype. Interestingly, recent studies proposed that besides its chemokine and opioid ligands, ACKR3 acts as a molecular rheostat for the proangiogenic peptide adrenomedullin (ADM).^12,13^ Indeed, *Ackr3* knockout recapitulates the *Adm* overexpression phenotype, and genetic reduction of *Adm* expression counterbalances lymphatic and cardiac abnormalities observed in *Ackr3* knockout mice.^12^

Adrenomedullin is a 52 amino acid (AA) peptide, acting as a vital paracrine factor to promote cardiac development, vasodilation, and formation of blood and lymph vessels.^14,15^ Due to its proangiogenic properties, ADM is also a key player in tumor growth.^16^ ADM belongs to calcitonin/calcitonin gene-related peptide (CGRP) family that also includes α-CGRP and β-CGRP, intermedin/adrenomedullin 2 (IMD/ADM2), amylin (AMY), and calcitonin (CT).^17^ ADM binds and activates the G-protein-coupled receptor (GPCR) calcitonin receptor-like receptor (CLR), which can only be exported to the cell surface upon heterodimerization with one of the three accessory membrane proteins called receptor activity modifying proteins (RAMPs).^18−20^ RAMP interactions also define the pharmacological profile of CLR. While a complex with either RAMP2 or RAMP3 generates a selective ADM receptor, dimerization with RAMP1 creates a receptor for CGRP with only low affinity for ADM.^17^

Adrenomedullin is generated through the proteolysis of a precursor molecule called proadrenomedullin (proADM), which also gives rise to the proadrenomedullin N-terminal 20 peptide (PAMP) (Figure 1A).^21,22^ PAMP is a 20 AA peptide involved in similar processes as ADM, but it differs in size and sequence and has no activity toward the ADM receptor complex CLR/RAMPs. Instead, the Mas-related G-protein-coupled receptor member X2 (MrgX2 or MRGPRX2) was proposed as the receptor for PAMP as well as for its endogenously processed form, PAMP-12, consisting of AAs 9–20.^23,24^ It is still unknown whether the observed physiological effects of PAMP rely exclusively on MrgX2 or on additional receptors. Although the vast majority of studies focus on ADM rather than on PAMP functions, both peptides are often found in the same regions and exert similar effects,^25,26^ suggesting that they may act in parallel. However, the roles and the receptors of PAMP are largely under-investigated.

**Figure 1:**
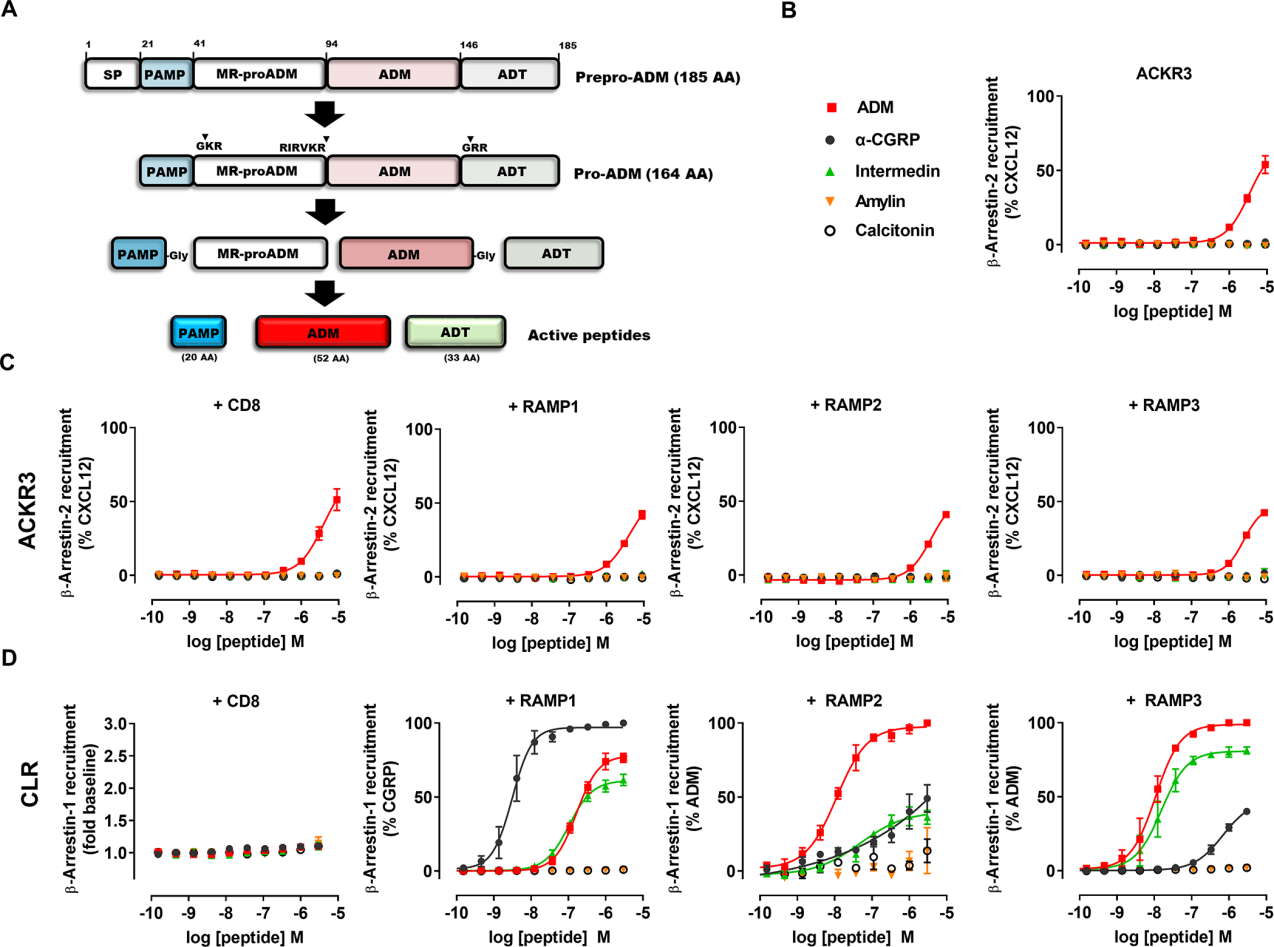
ADM is the only CGRP-family member with limited ACKR3 activity not influenced by RAMPs. (A) Schematic overview of preproADM processing into at least two active peptides (PAMP and ADM) and adrenotensin (ADT), whose bioactivity has to be confirmed. Amino acid (AA) motifs recognized by pro-protein convertases potentially involved during pro-ADM maturation are indicated. MR-proADM: midregional proadrenomedullin, SP: signal peptide. (B–D) Efficacy and potency of different CGRP family members in inducing β-arrestin recruitment toward ACKR3 (B and C) or CLR (D) in HEK cells in the absence of regulatory proteins (B), or in the presence of one of the three RAMPs or CD8 used as negative control protein (C and D) using NanoBiT technology. Results are expressed as percentage of full agonist response and represent the mean ± SEM of three independent experiments (*n* = 3).

Although important biological and genetic links have been established between ADM expression and ACKR3,^10,12^ the exact regulatory role of ACKR3 in ADM signaling, the pharmacological properties of ADM toward ACKR3 as well as the possible impact of ACKR3 on other proADM-derived peptides and ligands of the CGRP family have not been comprehensively assessed.^13,27^

In this study, we demonstrate that ADM is the only member of the CGRP family that activates ACKR3, with moderate micromolar-range activity. Remarkably, we found that PAMP, the second active peptide released during proADM maturation, has an activity toward ACKR3 that is comparable to ADM. Its truncated endogenous analog PAMP-12 especially shows a greater potency toward ACKR3 than ADM, which is comparable to the high-nanomolar range activity toward its previously identified receptor MrgX2. ACKR3 induces β-arrestin recruitment and drives PAMP-12 internalization, but in contrast to MrgX2, it does not induce classical G protein signaling or ERK phosphorylation. Our data suggest that the *ADM*-encoded PAMP-12 peptide is an additional endogenous ligand of ACKR3 and cast light on the potential role of PAMP-12, along with ADM, on the phenotypes observed in *Adm* knockout animals or overexpression experiments.

## Results and Discussion

### ADM Is the Only CGRP Family Member Showing Activity toward ACKR3

In order to characterize the activity and pharmacology of ADM toward ACKR3, we first measured its ability to induce β-arrestin-2 recruitment to ACKR3 using a nanoluciferase complementation-based assay (NanoBiT). We additionally included other structurally and functionally related peptides of the CGRP family, namely, α-CGRP, intermedin (IMD), amylin (AMY), and calcitonin (CT), to investigate the selectivity of ACKR3. Among these peptides, only ADM showed moderate activity toward ACKR3, triggering at the highest concentration tested (9 μM) about 50% of the maximum response observed with the full agonist CXCL12 (Figure 1B). No activity was detected with any of the other members of the CGRP family. However, although ACKR3 can heterodimerize with all three RAMP isoforms (Supplementary Figure 1), coexpression of ACKR3 with RAMPs did not improve its responsiveness to ADM, as already suggested in a recent study,^13^ or to any other CGRP family ligands (Figure 1C and Supplementary Table 1). In contrast, CLR activation was only observed upon coexpression of one of the three RAMP isoforms, as previously described^17,18^ (Figure 1D and Supplementary Table 1).

These results confirm that ADM is a weak agonist of ACKR3 and that its activity and pharmacology are not influenced by the presence of RAMPs.^13^ However, the apparent 300-fold lower ADM activity toward ACKR3 compared to CLR/RAMP2 or CLR/RAMP3 may question the physiological relevance of ACKR3 as an ADM receptor and suggests that the regulatory role of ACKR3 in the ADM signaling axis is either indirect, occurs in a particular microenvironment, or requires additional, so far unknown, accessory proteins.^28^

### proADM-Derived PAMP-12 Has a Stronger Potency toward ACKR3 than Mature ADM

**Figure 2:**
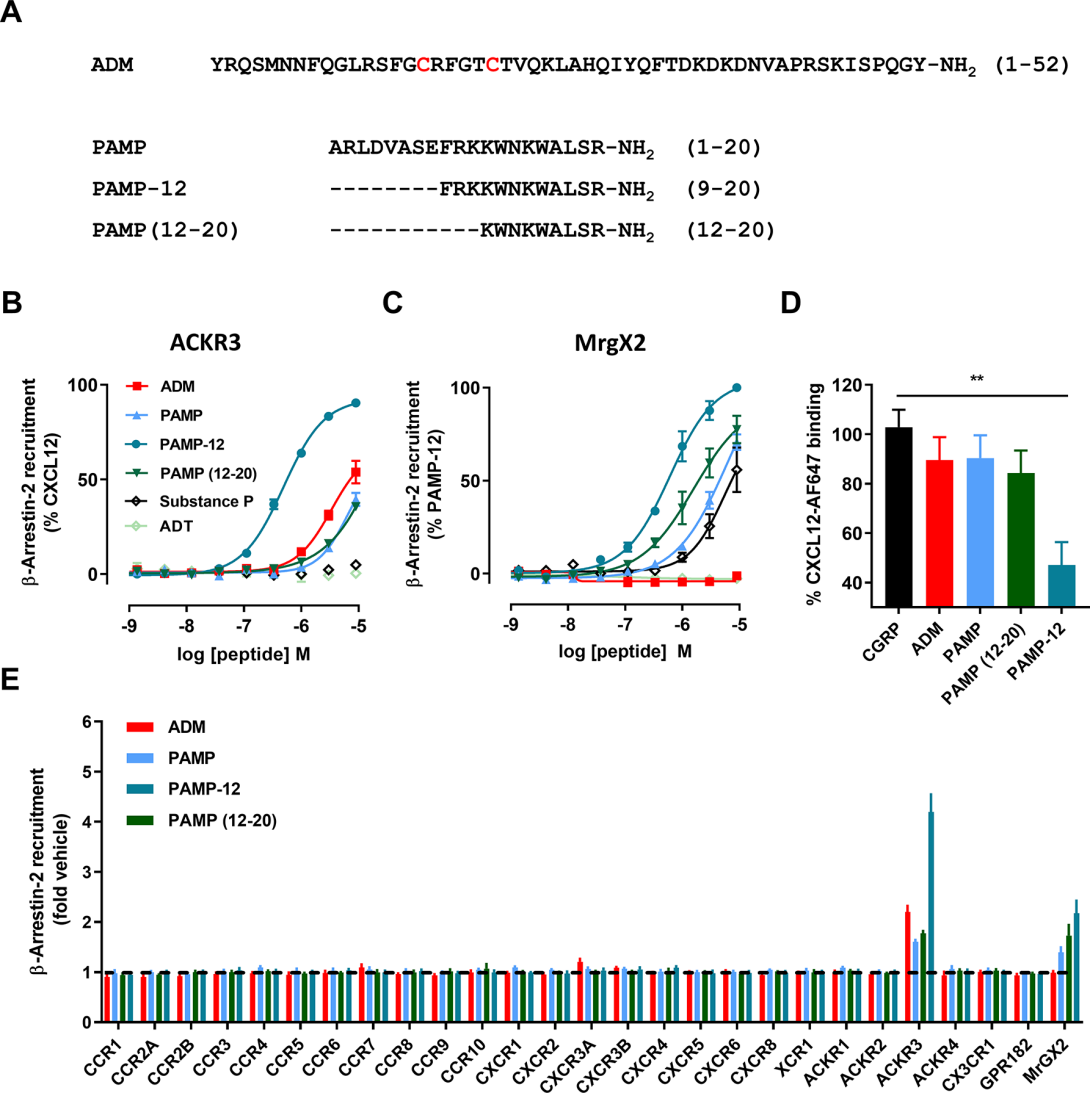
PAMP peptides have comparable activity toward ACKR3 and MrgX2 and no activity toward any other chemokine receptor. (A) Sequences of ADM and the three PAMP variants tested: full-length PAMP, comprising AAs 1–20; PAMP-12, comprising AAs 9–20; and PAMP(12–20), comprising the last 9 AAs of PAMP. For ADM, cysteine residues involved in a disulfide bridge forming a 4-residue intrapeptide arch are depicted in red. (B–C) Comparison of potency and efficacy of different active PAMP variants, ADM, ADT, and substance P in inducing β-arrestin-2 toward ACKR3 (B) or MrgX2 (C) in HEK cells, normalized to percent activity of their respective full agonists. (D) Binding competition of CGRP, ADM, and PAMP variants (9 μM) with AlexaFluor647-labeled CXCL12 (5 nM) on HEK-ACKR3 cells determined by flow cytometry. (E) Agonist activity of ADM and different PAMP variants (3 μM) toward all chemokine receptors, as well as the MrgX2 and GPR182 monitored in a β-arrestin-2 recruitment assay. Results are expressed as fold change over vehicle. For each receptor, an agonist chemokine (100 nM) listed in the IUPHAR repository of chemokine receptor ligands was used as the positive control. Results from B–E are represented as mean ± SEM of three independent experiments. **, *p* < 0.01 by one-way ANOVA with Bonferroni multiple comparison test.

Considering the strong biological link between *ACKR3* and *ADM*, we wondered whether ACKR3 might be activated by other peptides originating from the proADM precursor, namely, proadrenomedullin N-terminal 20 peptide (PAMP), and adrenotensin (ADT), a sparsely characterized peptide suggested to exert angiogenic activity on its own^29^ (Figure 1A). Surprisingly, while no activity of ADT could be detected, PAMP induced slightly lower β-arrestin-2 recruitment toward ACKR3 (EC_50_ > 10 μM) than did mature ADM (EC_50_ ≈ 5–10 μM) (Figure 2A,B). Examining further processed forms of PAMP, we found that PAMP-12, consisting of AAs 9–20 (FRKKWNKWALSR-NH_2_) (Figure 2A), showed a much greater potency (EC_50_ = 839 nM) toward ACKR3 compared to PAMP and ADM and acted as a full ACKR3 agonist for β-arrestin-2 recruitment (Figure 2B). PAMP-12 is often considered as the main active form of PAMP, since it shows stronger *in vivo* effects. In our assay it activated MrgX2 with higher potency (EC_50_ = 785 nM) than did the full-length PAMP (EC_50_ = 6.2 μM) (Figure 2C and Supplementary Table 1).^23,24,30^ PAMP(12–20), consisting of AAs 12–20 (KWNKWALSR-NH_2_), had a reduced potency (EC_50_ > 10 μM) toward ACKR3, comparable to that of full-length PAMP, indicating that the determinants for PAMP-12 activity lie within its N-terminal residues (FRK). These results were confirmed in a β-arrestin-1 recruitment assay (Supplementary Figure 2) and in a binding competition assay, where all identified peptides displaced CXCL12-AF647 from ACKR3, with PAMP-12 being by far the most potent competitor (Figure 2D). Of note, coexpression with RAMPs did not modify the activity of PAMP-derived peptides toward ACKR3, and no activity of PAMP peptides was detected on CLR or CLR/RAMP complexes (Supplementary Figure 2).

Importantly, while PAMP and PAMP(12–20) had reduced potency toward ACKR3 compared to that of their classical receptor MrgX2, PAMP-12 had equivalent potencies toward both receptors (Figure 2B,C and Supplementary Table 1). In agreement with the literature, PAMP-12 was also the most potent PAMP peptide toward MrgX2 in our β-arrestin-2 recruitment assay, although its apparent potency was lower than that described previously, which may be due to the different receptor activation readouts used.^23^ Noteworthy, ADM did not show any activity toward MrgX2 (Figure 2C) while substance P, another 11 AA (RPKPQQFFGLM-NH_2_) ligand of MrgX2, had no activity toward ACKR3 (Figure 2B), demonstrating that not all ligands are interchangeable between ACKR3 and MrgX2.

### ACKR3 Is the Only Chemokine Receptor Activated by proADM-Derived Peptides

The promiscuity of chemokines for their receptors is remarkable: Many chemokines bind to several receptors, while a single chemokine receptor can have multiple ligands. In order to evaluate the selectivity of the proADM-derived ligands for ACKR3, we screened ADM- and PAMP-derived peptides (PAMP, PAMP-12, and PAMP(12–20)) in a β-arrestin-2 recruitment assay toward all known classical and atypical human chemokine receptors. Our results show that ADM and PAMP-derived peptides are selective for ACKR3 and do not activate any of the other 24 chemokine receptors tested (Figure 2E), while MrgX2 is only activated by PAMP and its variants but not by mature ADM. Of note, no activity toward GPR182, the GPCR phylogenetically closest to ACKR3 and a debated adrenomedullin receptor,^31,32^ could be detected upon ligand treatment in this assay. These results indicate that ACKR3 is the only receptor with dual ADM–PAMP activation capacity.

### PAMP-12 SAR Analysis Pinpoints Different Key Residues for Activation of ACKR3 Compared to MrgX2

**Figure 3:**
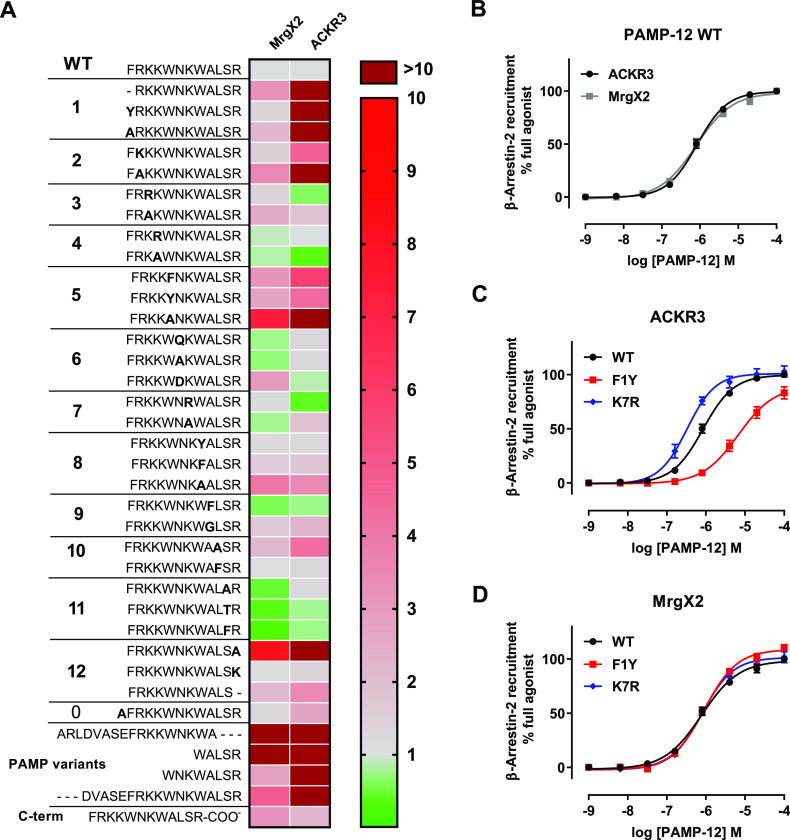
SAR analysis of PAMP-12 variants on ACKR3 and MrgX2. (A) Comparison of the impact of substitutions or truncations on the agonist activity of PAMP-12 toward ACKR3 and MrgX2. The agonist activity of each variant was evaluated in a β-arrestin-2 recruitment assay in HEK cells and expressed in a heat map as fold change in EC_50_ values with respect to wild-type PAMP-12. Four variants of PAMP were also included: PAMP(1–17), PAMP(16–20), PAMP(13–20), and PAMP(4–20). (B–D) Comparison of potency and efficacy of PAMP-12 (B) and its variants bearing mutations F1Y or K7R in inducing β-arrestin-2 recruitment to ACKR3 (C) and MrgX2 (D) in HEK cells. Results represent the mean (A) or mean ± SEM (B−D) of three independent experiments (*n* = 3). The corresponding pEC_50_ values are available in Supplementary Table 2.

In order to gain a deeper insight into the activation mechanism of ACKR3 by this new class of ligands, we performed a comparative structure–activity relationship (SAR) analysis using as the basis the most active peptide, PAMP-12, that shows comparable potencies on the two receptors (Figure 3A,B). In addition to a complete alanine scan, we compared the impact of different single amino acid substitutions and N-terminal extensions of PAMP-12, as well as several truncations of PAMP on the activation of ACKR3 and MrgX2, using β-arrestin-2 recruitment as readout (Figure 3A and Supplementary Table 2).

This analysis revealed that although a similar trend in potency shift toward the two receptors was observed for modifications at multiple positions, including W5, W8, L10, or R12, important differences could be highlighted. For instance, a phenylalanine at the first position of the peptide is required for a strong activity toward ACKR3 (Figure 3A and C). This is in stark contrast to MrgX2 (Figure 3D), but it is in full agreement with what we recently found in an adrenorphin SAR study, where the opposite Y1F mutation led to a 10-fold enhancement in potency of the peptide toward ACKR3^8^ and reminiscent of the phenylalanine at position 1 in CXCL11. Similarly, the SAR analysis revealed that R2 is crucial for PAMP-12 activity toward ACKR3. Together, these observations are in line with the previously measured differences in potency between PAMP-12 and PAMP(12–20) and further confirm that the determinants for PAMP-12 activity toward ACKR3 mainly lie within its N-terminal residues. Of all modifications, only lysine substitutions K3R, K4A, and K7R improved the potency toward ACKR3, while they were neutral for MrgX2. This also aligns with the previously reported adrenorphin SAR study, where the opposite R7K mutation was detrimental for ACKR3 activation^8^ and conservation of an arginine residue at position 7 or 8 within the N terminus of ACKR3-activating chemokines.^27^ Overall, these data demonstrate a high degree of similarity between PAMP, the opioid core and the N-terminal sequences of the ACKR3 chemokines probably reflecting a conserved binding mode and ACKR3 binding pocket occupancy. Of note, many mutations had only a minor impact on ACKR3 and MrgX2, pointing toward a high propensity for activation of both receptors toward PAMP-12. However, other truncated PAMP variants including PAMP(1–17), PAMP(16–20), PAMP(13–20), and PAMP(4–20) showed no activity toward ACKR3, highlighting some degree of selectivity of ACKR3 toward this class of ligands. Overall, this analysis shows that MrgX2 and ACKR3, while both showing ligand promiscuity, have somewhat different binding pockets for PAMP peptides.

### ACKR3 Mediates PAMP-12 Uptake without Inducing Signaling Events

**Figure 4:**
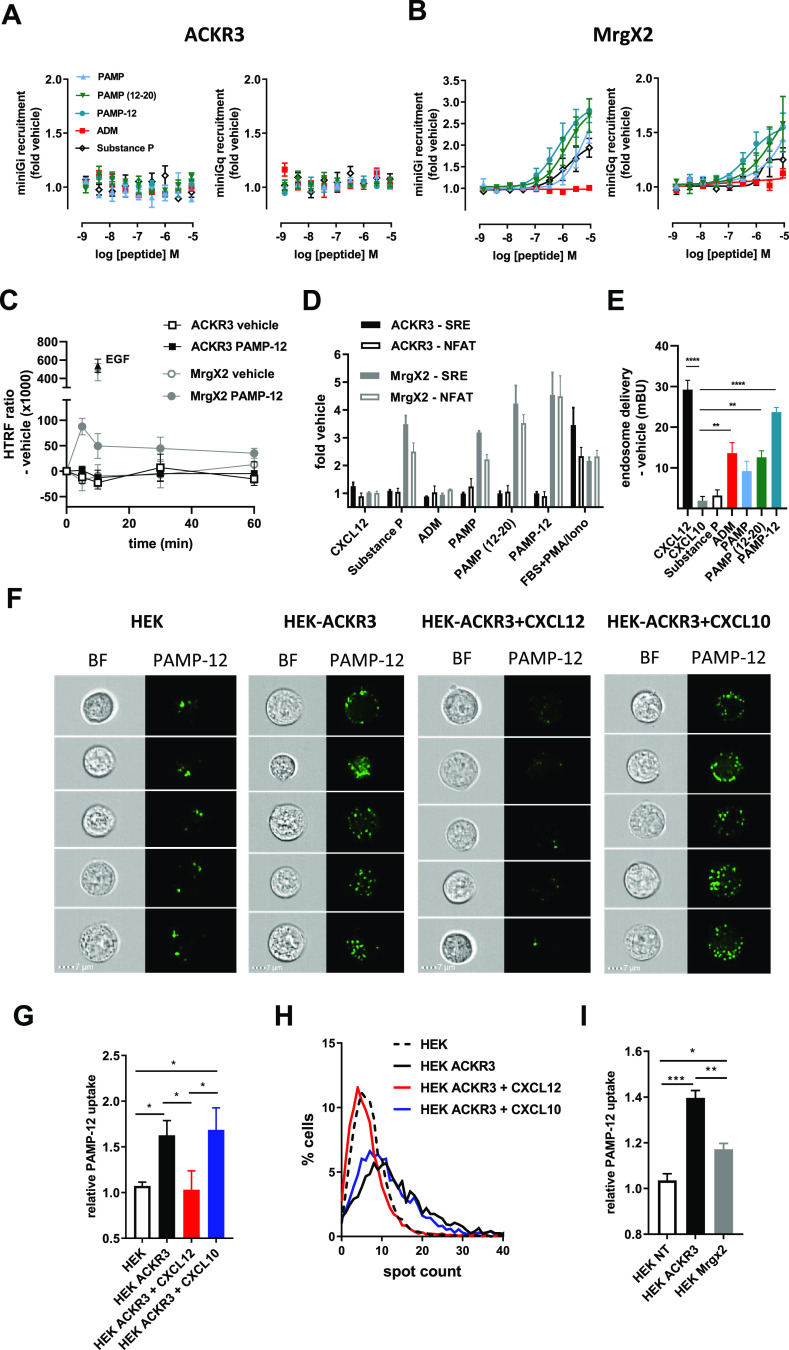
ACKR3 internalizes PAMP-12 without inducing G protein or ERK-signaling. (A, B) Comparison of miniGi and miniGq recruitment to ACKR3 (A) and MrgX2 (B) in response to ADM, substance P, and PAMP variants monitored in HEK cells using NanoBiT technology. (C) Kinetic analysis of ERK1/2 phosphorylation in HEK cells transfected with ACKR3- or MrgX2-encoding plasmids treated with vehicle or PAMP-12 (3 μM). EGF (100 nM) was used as positive control. (D) Activation of ERK (SRE) and Ca^2+^ (NFAT) signaling cascades in HEK cells expressing ACKR3 or MrgX2 in response to CXCL12 (300 nM), ADM, substance P, or PAMP variants (3 μM) or positive control (30 nM PMA, 10% FBS for SRE; 30 nM PMA, 1 μM ionomycin, 10% FBS for NFAT). (E) ACKR3 delivery to endosomes induced by peptides (3 μM) and chemokines (300 nM) monitored in HEK cells by NanoBRET using nanoluciferase-tagged β-arrestin-2 and mNeonGreen-tagged FYVE domain of endofin, which binds phosphatidylinositol 3-phosphate (PI3P) in early endosomes. Results are expressed in miliBRET units (mBU). (F–I) Uptake of fluorescently labeled PAMP-12 (PAMP-12-FAM, 3 μM). (F) Uptake of PAMP-12-FAM by ACKR3-positive or -negative HEK cells pretreated (or not) with CXCL12 or CXCL10 (200 nM) visualized by imaging flow cytometry. Five representative HEK or HEK-ACKR3 cells are shown. BF: brightfield. Scale bar: 7 μm. (G) PAMP-12-FAM uptake for conditions described in (F), quantified by mean fluorescence intensity (MFI) and normalized to signal obtained for nontransfected HEK cells. (H) Percentage of cells with a given number of distinguishable vesicle-like structures (spots) for conditions determined in (F). (I) PAMP-12-FAM uptake by HEK cells, transiently transfected with equal amounts of ACKR3 or MrgX2 encoding plasmids or an empty vector (NT) quantified by MFI. For all panels, results represent the mean ± SEM of at least three independent experiments (*n* ≥ 3) except for F and H, where one representative experiment of three independent repetitions is shown. *, *p* < 0.05; **, *p* < 0.01; ***, *p* < 0.001; and ****, *p* < 0.0001 by one-way ANOVA with Bonferroni (E) or Tuckey’s multiple comparison test (G and I).

The ability of ACKR3 to signal upon ligand binding is still highly debated and may be cell-type-dependent. While some studies reported signaling capacity for ACKR3, especially β-arrestin-dependent ERK phosphorylation,^33,34^ others suggested that ACKR3 acts as a non-signaling scavenger receptor.^8,35^ In order to assess the ability of ACKR3 to signal in response to proADM-derived peptides, we first explored the possibility that ACKR3 could couple to G proteins in response to ADM or PAMP variants by monitoring the miniGi and miniGq recruitment to the receptor in a nanoluciferase complementation-based assay. In contrast to MrgX2, for which all PAMP variants increased miniGi and miniGq interactions with the receptor in a concentration-dependent manner, we did not detect ligand-induced interactions between ACKR3 and miniGi or miniGq (Figure 4A,B). Furthermore, we did not observe any increase in ERK phosphorylation upon PAMP-12 treatment, or any activation of the MAPK/ERK-dependent serum response element (SRE) and of the calcium-dependent nuclear factor of activated T-cell response element (NFAT-RE) upon ADM or PAMP stimulation in ACKR3-transfected cells, in contrast to MrgX2-transfected cells (Figure 4C,D).

Our data suggest that ADM, PAMP, PAMP(12–20), and especially PAMP-12 can trigger β-arrestin recruitment to ACKR3 without inducing classical downstream signaling. In line with these data, recent studies proposed ACKR3 as a scavenging receptor for ADM, reducing ADM levels to regulate its activity.^12,13^ We therefore wondered whether ACKR3 might play a similar role for PAMP peptides. To this end, using nanoluciferase bioluminescence resonance energy transfer (NanoBRET), we first investigated whether the ACKR3/β-arrestin complex is internalized and delivered to early endosomes upon receptor activation by PAMP peptides.^36^ We observed a robust BRET signal upon treatment of ACKR3-expressing cells with CXCL12, ADM, and PAMP peptides, but not with negative controls CXCL10 or substance P, indicative of a specific delivery of the ligands to endosomes upon binding to ACKR3 (Figure 4E). Using imaging flow cytometry, we could also demonstrate that ACKR3 is able to internalize fluorescently labeled PAMP-12. We observed a clear intracellular accumulation of fluorescently labeled PAMP-12 after 45 min of stimulation of HEK-ACKR3 cells, with a notably higher number of distinguishable vesicle-like structures and a higher mean fluorescent intensity compared to those of naïve HEK cells (Figure 4F–H). Preincubation of HEK-ACKR3 cells with CXCL12, but not with the control chemokine CXCL10, reduced PAMP-12 accumulation to background levels, suggesting a specific ACKR3-driven uptake (Figure 4F–H). Moreover, despite a similar potency of PAMP-12 toward MrgX2 and ACKR3, MrgX2-positive cells showed significantly less peptide uptake than ACKR3-positive cells, underscoring the scavenging capacity of ACKR3 (Figure 4I). In conclusion, our data suggest that similar to chemokines and endogenous opioid peptides ACKR3 is able to reduce PAMP-12 availability by efficiently internalizing the peptide without inducing further signaling events.

Although PAMP peptides are involved in a variety of physiological processes like vasodilation, angiogenesis, cell migration, apoptosis, or degranulation of mast cells, not much is known about their pharmacological activity or their physiological regulation. Here, we describe a mechanism of PAMP-12 regulation via scavenging by ACKR3, which may restrain peptide availability for its signaling receptor MrgX2. Interestingly, although MrgX2 and ACKR3 are not closely related phylogenetically or functionally, both receptors were recently described to be activated by a variety of small endogenous opioid peptides such as dynorphins, making PAMPs their second shared family of ligands.^8,37^

Regardless of the regulatory role of ACKR3 toward ADM,^12^ its scavenging capacity for PAMP-12 may be superior and partly explain why ADM and PAMP, despite their common precursor, show different and non-equimolar tissue distribution in regions where ACKR3 is highly expressed.^2,38,39^ This spatiotemporal regulation of PAMP-12 may follow a mechanism similar to that described for CXCL12 and recently for opioid peptides and should be the focus of future *in vivo* investigations.^40^ Of note, even though *Ackr3* knockout in mice shows a similar phenotype as *Adm* overexpression, a recent study revealed that ADM^ΔPAMP/ΔPAMP^ mice, which carry the ADM but lack the PAMP-coding sequence, had no obvious anomalies, pointing toward a more complex (possibly dual ADM/PAMP-12) or non-homeostatic scavenging role of ACKR3 in the regulation of these peptides.^41^ Finally, in analogy to ACKR3 interplay with CXCR4 described to alter the signaling properties of the latter,^42−44^ the ability of ACKR3 to heterodimerize with MrgX2 and/or CLR receptors and modulate their activity remains to be investigated. Additionally, the trafficking and signaling properties of the two receptors may be indirectly affected by ACKR3’s interaction with RAMP3 that was recently reported to regulate its cycling.^13^ In conclusion, our study identifies ACKR3 as the first dual ADM/PAMP receptor and sheds light on the complex regulation of the availability of proADM-derived peptides.

## Materials and Methods

### Chemokines and Peptides

All chemokines were purchased from PeproTech. AlexaFluor647-labeled CXCL12 (CXCL12–AF647) was obtained from Almac. ADM and all other peptides from the CGRP family, as well as PAMP and PAMP(12–20) were acquired from Bachem. PAMP-12 was purchased from Phoenix Pharmaceuticals. PAMP-12 variants and (5-FAM)-labeled PAMP were synthesized by JPT. These peptides contain a free amine at the N-terminus and an amide group at the C-terminus.

### Cell Culture

HEK293T cells were purchased from ATCC and grown in DMEM supplemented with 10% fetal bovine serum (FBS) and penicillin/streptomycin (100 units/mL and 100 μg/mL). HEK293T cells stably expressing ACKR3 (HEK-ACKR3) were generated by transfection with a pIRES vector encoding human ACKR3 and maintained under puromycin (5 μg/mL) selective pressure. Cells were regularly tested for mycoplasma contamination.

### Binding Competition Assay

The assay was performed as previously described.^27,45^ In brief, HEK-ACKR3 cells were distributed into 96-well plates (2 × 10^5^ cells/well) and incubated with a mixture of CXCL12–AF647 (5 nM) and unlabeled peptides at indicated concentrations in FACS buffer (PBS, 1% BSA, 0.1% NaN_3_) for 90 min on ice. After two washing steps, the cells were incubated for 30 min at 4 °C with Zombie Green viability dye (BioLegend). After two washing steps, the cells were resuspended in FACS buffer and mean fluorescence intensity (MFI) was measured from 10 000 gated cells using a BD LSR Fortessa flow cytometer. The signal obtained for CXCL12–AF647 in the absence of unlabeled ligands was defined as 100% binding, and signal for CXCL12–AF647 in the presence of 1 μM unlabeled CXCL12 was set to 0%.

### Nanoluciferase Complementation-Based Assay (NanoBiT)

Ligand-induced recruitment of β-arrestin, miniGi or miniGq proteins (engineered GTPase domain of Gα subunit)^46,47^ to the receptors was monitored using NanoBiT technology (Promega), as previously described.^27,48^ HEK293T cells (5 × 10^6^ cells) were seeded in 10 cm dishes, and 24 h later, the cells were cotransfected with pNBe plasmids encoding the receptor C-terminally fused to SmBiT and β-arrestin, miniGi, or miniGq N-terminally fused to LgBiT. After 24 h, the cells were detached and incubated for 25 min at 37 °C with Nano-Glo Live Cell substrate diluted 200-fold, distributed into white 96-well plates (1 × 10^5^ cells/well), and treated with the indicated concentrations of peptides. Luminescence was recorded during 20 min with a Mithras LB940 luminometer (Berthold Technologies). For the concentration–response curves, the signal recorded with a saturating concentration of full agonist for each receptor was set as 100%. For receptor screening experiments, results were expressed as fold vehicle, and an agonist chemokine (100 nM) listed in the IUPHAR repository of chemokine receptor ligands was included as a positive control for each receptor.

### Nanoluciferase Bioluminescence Resonance Energy Transfer (NanoBRET)

Ligand-induced receptor–arrestin delivery to endosomes was monitored by NanoBRET. In brief, 5 × 10^6^ HEK293T cells were seeded in 10 cm dishes, and 24 h later, the cells were cotransfected with plasmids encoding ACKR3, β-arrestin-2 N-terminally tagged with nanoluciferase and the FYVE domain of endofin, interacting with phosphatidylinositol 3-phosphate (PI3P) in early endosomes,^36,49^ N-terminally tagged with mNeonGreen. After 24 h, the cells were detached and distributed into black 96-well plates (1 × 10^5^ cells/well) and treated with saturating concentrations of ligands (3 μM for peptides or 300 nM for chemokines). After 30 min of incubation at 37 °C, coelenterazine H (10 μM) was added, and donor emission (460 nm) and acceptor emission (535 nm) were immediately measured on a Mithras LB940 plate reader (Berthold Technologies).

For receptor dimerization experiments, HEK293T cells were seeded in a 12-well plate (5 × 10^5^ cells/well). After 24 h, the cells were transfected with 5 ng of donor-encoding pNLF vector (RAMP, CD8, ACKR3, or CXCR4 C-terminally tagged with nanoluciferase) and increasing concentrations of acceptor-encoding pNeonGreen vector (ACKR3, CXCR4, or CLR C-terminally tagged with mNeonGreen). An empty pcDNA3.1 vector was added to the different transfection mixes in order to maintain a constant total amount of DNA. At 24 h post-transfection, the cells were detached and seeded in black 96-well plates (1 × 10^5^ cells/well). The signal of mNeonGreen was first quantified (excitation, 485 nm; emission, 535 nm) and used to determine the acceptor/donor ratio. After coelenterazine H (10 μM) addition, donor emission (460 nm) and acceptor emission (535 nm) were immediately measured on a Mithras LB940 plate reader (Berthold Technologies). BRET ratios were plotted against the determined acceptor/donor ratio, and the data were fitted using a nonlinear regression equation for one site-specific binding.

### Inducible Nanoluciferase Reporter Gene Transcription Assays

Activation of the MAPK/ERK signaling pathway was evaluated using an SRE nanoluciferase reporter assay. Activation of calcium-dependent signaling pathways was evaluated using an NFAT-RE nanoluciferase reporter assay. In brief, 6 × 10^6^ HEK293T cells were seeded in 10 cm dishes, and 24 h later, the cells were cotransfected with a pcDNA3.1 encoding either ACKR3 or MrgX2 and pNanoLuc/SRE or pNanoLuc/NFAT-RE vectors (Promega) containing the nanoluciferase gene downstream of an SRE or NFAT-RE. After 24 h, the cells were detached and seeded in white 96-well plates (1 × 10^5^ cells/well). After 24 h, the medium was replaced by phenol-free DMEM, and after 2 h incubation, chemokines, peptides, or positive control (30 nM phorbol 12-myristate 13-acetate (PMA) + 10% FBS with or without 1 μM ionomycin for NFAT-RE and SRE, respectively) were added. After 6 h (SRE) or 8 h (NFAT-RE), Nano-Glo Live Cell substrate (Promega) was added, and the luminescence was read during 20 min on a Mithras LB940 plate reader (Berthold Technologies).

### HTRF-Based Determination of ERK1/2 Phosphorylation

An HTRF-based phospho-ERK1/2 (extracellular signal regulated kinases 1 and 2) assay was performed using the phospho-ERK1/2 (Thr202/Tyr204) cellular kit (Cisbio International). In brief, 6 × 10^6^ HEK293T cells were seeded in 10 cm dishes and transfected 24 h later with pcDNA3.1 plasmid encoding ACKR3 or MrgX2. At 24 h post-transfection, the cells were detached and seeded in 96-well plates (1 × 10^5^ cells/well). After 24 h, the cell culture medium was replaced with phenol-free DMEM, and after 90 min of incubation, the cells were stimulated with PAMP-12 (3 μM), vehicle, or epidermal growth factor (EGF, 100 nM) as positive control for the indicated time intervals. Supernatants were replaced with the provided lysis buffer, and 45 min later, the lysates were transferred to a white 384-well plate. After a 2 h of incubation with pERK1/2-specific antibodies conjugated to Eu^3+^-cryptate donor and d2 acceptor at the recommended dilutions, the HTRF signal was measured on a Tecan GENios pro plate reader equipped with a 340 nm excitation filter and 612 ± 10 nm (donor) and 670 ± 25 nm (acceptor) emission filters.

### Visualization of PAMP-12-FAM Uptake by Imaging Flow Cytometry

HEK293T or HEK-ACKR3 cells were harvested in Opti-MEM and distributed into 96-well plates (3 × 10^5^ cells/well). After a 15 min of incubation at 37 °C with CXCL10, CXCL12, or Opti-MEM only, FAM-labeled PAMP-12 was added to a final concentration of 3 μM and incubated for 45 min at 37 °C; then the cells were washed twice with FACS buffer. For comparison of labeled PAMP-12 uptake by ACKR3 or MrgX2, 6 × 10^6^ HEK293T cells were seeded in 10 cm dishes and transfected 24 h later with 4 μg of pcDNA3.1 plasmid encoding ACKR3 or MrgX2. At 24 h post-transfection, the cells were harvested and treated as described above. Dead cells were excluded using Zombie NIR viability dye (BioLegend). Images of 1 × 10^4^ in-focus, living single cells were acquired with an ImageStream Mark II imaging flow cytometer (Amnis) equipped with an extended depth of field (EDF) module and using 60× magnification. Samples were analyzed using Ideas6.2 software. The number of spots per cell was determined using a mask-based software wizard.

### Data and Statistical Analysis

Concentration–response curves were fitted to the four-parameter Hill equation using an iterative, least-squares method (GraphPad Prism version 8.0.1). All curves were fitted to data points generated from the mean of at least three independent experiments. Statistical tests, i.e., ordinary one-way ANOVA and post hoc analysis were performed with GraphPad Prism 8.0.1. The *p*-values are indicated as follows: *, *p* < 0.05; **, *p* < 0.01; ***, *p* < 0.001; and ****, *p* < 0.0001.

## Supporting Information Available

Dimerization assays between RAMPs and ACKR3, CXCR4, and CLR using NanoBRET, PAMP-mediated beta-arrestin recruitment to ACKR3 and CLR in the presence or absence of RAMPs, sequences of peptides used in this study and their potencies toward ACKR3, MrgX2, and CLR/RAMP (PDF)

## References

[ref1] Bachelerie F.; Ben-Baruch A.; Burkhardt A. M.; Combadiere C.; Farber J. M.; Graham G. J.; Horuk R.; Sparre-Ulrich A. H.; Locati M.; Luster A. D.; Mantovani A.; Matsushima K.; Murphy P. M.; Nibbs R.; Nomiyama H.; Power C. A.; Proudfoot A. E.; Rosenkilde M. M.; Rot A.; Sozzani S.; Thelen M.; Yoshie O.; Zlotnik A. (2014) International Union of Basic and Clinical Pharmacology. [corrected]. LXXXIX. Update on the extended family of chemokine receptors and introducing a new nomenclature for atypical chemokine receptors. Pharmacol. Rev. 66 (1), 1–79. 10.1124/pr.113.007724. 24218476 PMC3880466

[ref2] Regard J. B.; Sato I. T.; Coughlin S. R. (2008) Anatomical profiling of G protein-coupled receptor expression. Cell 135 (3), 561–71. 10.1016/j.cell.2008.08.040. 18984166 PMC2590943

[ref3] Berahovich R. D.; Zabel B. A.; Lewen S.; Walters M. J.; Ebsworth K.; Wang Y.; Jaen J. C.; Schall T. J. (2014) Endothelial expression of CXCR7 and the regulation of systemic CXCL12 levels. Immunology 141 (1), 111–22. 10.1111/imm.12176. 24116850 PMC3893854

[ref4] Burns J. M.; Summers B. C.; Wang Y.; Melikian A.; Berahovich R.; Miao Z.; Penfold M. E.; Sunshine M. J.; Littman D. R.; Kuo C. J.; Wei K.; McMaster B. E.; Wright K.; Howard M. C.; Schall T. J. (2006) A novel chemokine receptor for SDF-1 and I-TAC involved in cell survival, cell adhesion, and tumor development. J. Exp. Med. 203 (9), 2201–13. 10.1084/jem.20052144. 16940167 PMC2118398

[ref5] Szpakowska M.; Dupuis N.; Baragli A.; Counson M.; Hanson J.; Piette J.; Chevigne A. (2016) Human herpesvirus 8-encoded chemokine vCCL2/vMIP-II is an agonist of the atypical chemokine receptor ACKR3/CXCR7. Biochem. Pharmacol. 114, 14–21. 10.1016/j.bcp.2016.05.012. 27238288

[ref6] Alampour-Rajabi S.; El Bounkari O.; Rot A.; Muller-Newen G.; Bachelerie F.; Gawaz M.; Weber C.; Schober A.; Bernhagen J. (2015) MIF interacts with CXCR7 to promote receptor internalization, ERK1/2 and ZAP-70 signaling, and lymphocyte chemotaxis. FASEB J. 29 (11), 4497–511. 10.1096/fj.15-273904. 26139098

[ref7] Ikeda Y.; Kumagai H.; Skach A.; Sato M.; Yanagisawa M. (2013) Modulation of circadian glucocorticoid oscillation via adrenal opioid-CXCR7 signaling alters emotional behavior. Cell 155 (6), 1323–36. 10.1016/j.cell.2013.10.052. 24315101 PMC3934808

[ref8] Meyrath M.; Szpakowska M.; Zeiner J.; Massotte L.; Merz M. P.; Benkel T.; Simon K.; Ohnmacht J.; Turner J. D.; Kruger R.; Seutin V.; Ollert M.; Kostenis E.; Chevigne A. (2020) The atypical chemokine receptor ACKR3/CXCR7 is a broad-spectrum scavenger for opioid peptides. Nat. Commun. 11 (1), 3033 10.1038/s41467-020-16664-0. 32561830 PMC7305236

[ref9] Quinn K. E.; Mackie D. I.; Caron K. M. (2018) Emerging roles of atypical chemokine receptor 3 (ACKR3) in normal development and physiology. Cytokine+ 109, 17–23. 10.1016/j.cyto.2018.02.024. 29903572 PMC6005205

[ref10] Sierro F.; Biben C.; Martinez-Munoz L.; Mellado M.; Ransohoff R. M.; Li M.; Woehl B.; Leung H.; Groom J.; Batten M.; et al. (2007) Disrupted cardiac development but normal hematopoiesis in mice deficient in the second CXCL12/SDF-1 receptor, CXCR7. Proc. Natl. Acad. Sci. U. S. A. 104 (37), 14759–14764. 10.1073/pnas.0702229104. 17804806 PMC1976222

[ref11] Yu S.; Crawford D.; Tsuchihashi T.; Behrens T. W.; Srivastava D. (2011) The chemokine receptor CXCR7 functions to regulate cardiac valve remodeling. Dev. Dyn. 240 (2), 384–93. 10.1002/dvdy.22549. 21246655 PMC3079332

[ref12] Klein K. R.; Karpinich N. O.; Espenschied S. T.; Willcockson H. H.; Dunworth W. P.; Hoopes S. L.; Kushner E. J.; Bautch V. L.; Caron K. M. (2014) Decoy receptor CXCR7 modulates adrenomedullin-mediated cardiac and lymphatic vascular development. Dev. Cell 30 (5), 528–40. 10.1016/j.devcel.2014.07.012. 25203207 PMC4166507

[ref13] Mackie D. I.; Nielsen N. R.; Harris M.; Singh S.; Davis R. B.; Dy D.; Ladds G.; Caron K. M. (2019) RAMP3 determines rapid recycling of atypical chemokine receptor-3 for guided angiogenesis. Proc. Natl. Acad. Sci. U. S. A. 116 (48), 24093–24099. 10.1073/pnas.1905561116. 31712427 PMC6883789

[ref14] Caron K. M.; Smithies O. (2001) Extreme hydrops fetalis and cardiovascular abnormalities in mice lacking a functional Adrenomedullin gene. Proc. Natl. Acad. Sci. U. S. A. 98 (2), 615–9. 10.1073/pnas.98.2.615. 11149956 PMC14636

[ref15] Kitamura K.; Kangawa K.; Kawamoto M.; Ichiki Y.; Nakamura S.; Matsuo H.; Eto T. (1993) Adrenomedullin: a novel hypotensive peptide isolated from human pheochromocytoma. Biochem. Biophys. Res. Commun. 192 (2), 553–60. 10.1006/bbrc.1993.1451. 8387282

[ref16] Zudaire E.; Martinez A.; Cuttitta F. (2003) Adrenomedullin and cancer. Regul. Pept. 112 (1–3), 175–83. 10.1016/S0167-0115(03)00037-5. 12667640

[ref17] Hay D. L.; Garelja M. L.; Poyner D. R.; Walker C. S. (2018) Update on the pharmacology of calcitonin/CGRP family of peptides: IUPHAR Review 25. Br. J. Pharmacol. 175 (1), 3–17. 10.1111/bph.14075. 29059473 PMC5740251

[ref18] McLatchie L. M.; Fraser N. J.; Main M. J.; Wise A.; Brown J.; Thompson N.; Solari R.; Lee M. G.; Foord S. M. (1998) RAMPs regulate the transport and ligand specificity of the calcitonin-receptor-like receptor. Nature 393 (6683), 333–9. 10.1038/30666. 9620797

[ref19] Heroux M.; Breton B.; Hogue M.; Bouvier M. (2007) Assembly and signaling of CRLR and RAMP1 complexes assessed by BRET. Biochemistry 46 (23), 7022–33. 10.1021/bi0622470. 17503773

[ref20] Heroux M.; Hogue M.; Lemieux S.; Bouvier M. (2007) Functional calcitonin gene-related peptide receptors are formed by the asymmetric assembly of a calcitonin receptor-like receptor homo-oligomer and a monomer of receptor activity-modifying protein-1. J. Biol. Chem. 282 (43), 31610–20. 10.1074/jbc.M701790200. 17785463

[ref21] Kitamura K.; Sakata J.; Kangawa K.; Kojima M.; Matsuo H.; Eto T. (1993) Cloning and characterization of cDNA encoding a precursor for human adrenomedullin. Biochem. Biophys. Res. Commun. 194 (2), 720–5. 10.1006/bbrc.1993.1881. 7688224

[ref22] Kim W.; Essalmani R.; Szumska D.; Creemers J. W.; Roebroek A. J.; D’Orleans-Juste P.; Bhattacharya S.; Seidah N. G.; Prat A. (2012) Loss of endothelial furin leads to cardiac malformation and early postnatal death. Mol. Cell. Biol. 32 (17), 3382–91. 10.1128/MCB.06331-11. 22733989 PMC3422005

[ref23] Kamohara M.; Matsuo A.; Takasaki J.; Kohda M.; Matsumoto M.; Matsumoto S.; Soga T.; Hiyama H.; Kobori M.; Katou M. (2005) Identification of MrgX2 as a human G-protein-coupled receptor for proadrenomedullin N-terminal peptides. Biochem. Biophys. Res. Commun. 330 (4), 1146–52. 10.1016/j.bbrc.2005.03.088. 15823563

[ref24] Kuwasako K.; Kitamura K.; Ishiyama Y.; Washimine H.; Kato J.; Kangawa K.; Eto T. (1997) Purification and characterization of PAMP-12 (PAMP[9–20]) in porcine adrenal medulla as a major endogenous biologically active peptide. FEBS Lett. 414 (1), 105–10. 10.1016/S0014-5793(97)00971-X. 9305741

[ref25] Andreis P. G.; Tortorella C.; Mazzocchi G.; Nussdorfer G. G. (1998) Proadrenomedullin N-terminal 20 peptide inhibits aldosterone secretion of human adrenocortical and Conn’s adenoma cells: comparison with adrenomedullin effect. J. Clin. Endocrinol. Metab. 83 (1), 253–257. 10.1210/jcem.83.1.4517. 9435451

[ref26] Eto T. (2001) A review of the biological properties and clinical implications of adrenomedullin and proadrenomedullin N-terminal 20 peptide (PAMP), hypotensive and vasodilating peptides. Peptides 22 (11), 1693–711. 10.1016/S0196-9781(01)00513-7. 11754955

[ref27] Szpakowska M.; Meyrath M.; Reynders N.; Counson M.; Hanson J.; Steyaert J.; Chevigne A. (2018) Mutational analysis of the extracellular disulphide bridges of the atypical chemokine receptor ACKR3/CXCR7 uncovers multiple binding and activation modes for its chemokine and endogenous non-chemokine agonists. Biochem. Pharmacol. 153, 299–309. 10.1016/j.bcp.2018.03.007. 29530506

[ref28] Magalhaes A. C.; Dunn H.; Ferguson S. S. (2012) Regulation of GPCR activity, trafficking and localization by GPCR-interacting proteins. Br. J. Pharmacol. 165 (6), 1717–1736. 10.1111/j.1476-5381.2011.01552.x. 21699508 PMC3372825

[ref29] Okumura A.; Takahashi E.; Unoki-Kubota H.; Kaburagi Y. (2016) A novel angiogenic peptide, DeltaADT: A truncated adrenotensin peptide revealed by secretory peptidome analysis of human retinal pericytes. BioSci. Trends 10 (6), 500–506. 10.5582/bst.2016.01189. 27840371

[ref30] Kobayashi H.; Yamamoto R.; Kitamura K.; Kuwasako K.; Minami S.; Yanagita T.; Shiraishi S.; Yokoo H.; Eto T.; Wada A. (2001) Selective inhibition of nicotinic cholinergic receptors by proadrenomedullin N-terminal 12 peptide in bovine adrenal chromaffin cells. Mol. Brain Res. 87 (2), 175–83. 10.1016/S0169-328X(01)00011-0. 11245919

[ref31] Kennedy S. P.; Sun D.; Oleynek J. J.; Hoth C. F.; Kong J.; Hill R. J. (1998) Expression of the rat adrenomedullin receptor or a putative human adrenomedullin receptor does not correlate with adrenomedullin binding or functional response. Biochem. Biophys. Res. Commun. 244 (3), 832–7. 10.1006/bbrc.1998.8349. 9535752

[ref32] Ramachandran V.; Arumugam T.; Langley R.; Hwang R. F.; Vivas-Mejia P.; Sood A. K.; Lopez-Berestein G.; Logsdon C. D. (2009) The ADMR receptor mediates the effects of adrenomedullin on pancreatic cancer cells and on cells of the tumor microenvironment. PLoS One 4 (10), e7502 10.1371/journal.pone.0007502. 19847298 PMC2760778

[ref33] Heuninck J.; Perpina Viciano C.; Isbilir A.; Caspar B.; Capoferri D.; Briddon S. J.; Durroux T.; Hill S. J.; Lohse M. J.; Milligan G.; Pin J. P.; Hoffmann C. (2019) Context-Dependent Signaling of CXC Chemokine Receptor 4 and Atypical Chemokine Receptor 3. Mol. Pharmacol. 96 (6), 778–793. 10.1124/mol.118.115477. 31092552

[ref34] Rajagopal S.; Kim J.; Ahn S.; Craig S.; Lam C. M.; Gerard N. P.; Gerard C.; Lefkowitz R. J. (2010) Beta-arrestin- but not G protein-mediated signaling by the “decoy” receptor CXCR7. Proc. Natl. Acad. Sci. U. S. A. 107 (2), 628–32. 10.1073/pnas.0912852107. 20018651 PMC2818968

[ref35] Naumann U.; Cameroni E.; Pruenster M.; Mahabaleshwar H.; Raz E.; Zerwes H. G.; Rot A.; Thelen M. (2010) CXCR7 functions as a scavenger for CXCL12 and CXCL11. PLoS One 5 (2), e9175 10.1371/journal.pone.0009175. 20161793 PMC2820091

[ref36] Namkung Y.; Le Gouill C.; Lukashova V.; Kobayashi H.; Hogue M.; Khoury E.; Song M.; Bouvier M.; Laporte S. A. (2016) Monitoring G protein-coupled receptor and beta-arrestin trafficking in live cells using enhanced bystander BRET. Nat. Commun. 7, 12178 10.1038/ncomms12178. 27397672 PMC4942582

[ref37] Lansu K.; Karpiak J.; Liu J.; Huang X. P.; McCorvy J. D.; Kroeze W. K.; Che T.; Nagase H.; Carroll F. I.; Jin J.; Shoichet B. K.; Roth B. L. (2017) In silico design of novel probes for the atypical opioid receptor MRGPRX2. Nat. Chem. Biol. 13 (5), 529–536. 10.1038/nchembio.2334. 28288109 PMC5391270

[ref38] Montuenga L. M.; Burrell M. A.; Garayoa M.; Llopiz D.; Vos M.; Moody T.; Garcia-Ros D.; Martinez A.; Villaro A. C.; Elsasser T.; Cuttitta F. (2000) Expression of proadrenomedullin derived peptides in the mammalian pituitary: co-localization of follicle stimulating hormone and proadrenomedullin N-20 terminal peptide-like peptide in the same secretory granules of the gonadotropes. J. Neuroendocrinol. 12 (7), 607–17. 10.1046/j.1365-2826.2000.00468.x. 10849205

[ref39] Lopez J.; Cuesta N.; Martinez A.; Montuenga L.; Cuttitta F. (1999) Proadrenomedullin N-terminal 20 peptide (PAMP) immunoreactivity in vertebrate juxtaglomerular granular cells identified by both light and electron microscopy. Gen. Comp. Endocrinol. 116 (2), 192–203. 10.1006/gcen.1999.7365. 10562449

[ref40] Boldajipour B.; Mahabaleshwar H.; Kardash E.; Reichman-Fried M.; Blaser H.; Minina S.; Wilson D.; Xu Q.; Raz E. (2008) Control of chemokine-guided cell migration by ligand sequestration. Cell 132 (3), 463–73. 10.1016/j.cell.2007.12.034. 18267076

[ref41] Matson B. C.; Li M.; Trincot C. E.; Blakeney E. S.; Pierce S. L.; Caron K. M. (2019) Genetic loss of proadrenomedullin N-terminal 20 peptide (PAMP) in mice is compatible with survival. Peptides 112, 96–100. 10.1016/j.peptides.2018.11.005. 30537525 PMC6362461

[ref42] Decaillot F. M.; Kazmi M. A.; Lin Y.; Ray-Saha S.; Sakmar T. P.; Sachdev P. (2011) CXCR7/CXCR4 heterodimer constitutively recruits beta-arrestin to enhance cell migration. J. Biol. Chem. 286 (37), 32188–97. 10.1074/jbc.M111.277038. 21730065 PMC3173186

[ref43] Levoye A.; Balabanian K.; Baleux F.; Bachelerie F.; Lagane B. (2009) CXCR7 heterodimerizes with CXCR4 and regulates CXCL12-mediated G protein signaling. Blood 113 (24), 6085–93. 10.1182/blood-2008-12-196618. 19380869

[ref44] Coggins N. L.; Trakimas D.; Chang S. L.; Ehrlich A.; Ray P.; Luker K. E.; Linderman J. J.; Luker G. D. (2014) CXCR7 controls competition for recruitment of beta-arrestin 2 in cells expressing both CXCR4 and CXCR7. PLoS One 9 (6), e98328 10.1371/journal.pone.0098328. 24896823 PMC4045718

[ref45] Szpakowska M.; Nevins A. M.; Meyrath M.; Rhainds D.; D’Huys T.; Guite-Vinet F.; Dupuis N.; Gauthier P. A.; Counson M.; Kleist A.; St-Onge G.; Hanson J.; Schols D.; Volkman B. F.; Heveker N.; Chevigne A. (2018) Different contributions of chemokine N-terminal features attest to a different ligand binding mode and a bias towards activation of ACKR3/CXCR7 compared with CXCR4 and CXCR3. Br. J. Pharmacol. 175 (9), 1419–1438. 10.1111/bph.14132. 29272550 PMC5900987

[ref46] Carpenter B.; Nehme R.; Warne T.; Leslie A. G.; Tate C. G. (2016) Structure of the adenosine A(2A) receptor bound to an engineered G protein. Nature 536 (7614), 104–7. 10.1038/nature18966. 27462812 PMC4979997

[ref47] Wan Q.; Okashah N.; Inoue A.; Nehme R.; Carpenter B.; Tate C. G.; Lambert N. A. (2018) Mini G protein probes for active G protein-coupled receptors (GPCRs) in live cells. J. Biol. Chem. 293 (19), 7466–7473. 10.1074/jbc.RA118.001975. 29523687 PMC5949987

[ref48] Dixon A. S.; Schwinn M. K.; Hall M. P.; Zimmerman K.; Otto P.; Lubben T. H.; Butler B. L.; Binkowski B. F.; Machleidt T.; Kirkland T. A.; Wood M. G.; Eggers C. T.; Encell L. P.; Wood K. V. (2016) NanoLuc Complementation Reporter Optimized for Accurate Measurement of Protein Interactions in Cells. ACS Chem. Biol. 11 (2), 400–8. 10.1021/acschembio.5b00753. 26569370

[ref49] Schink K. O.; Raiborg C.; Stenmark H. (2013) Phosphatidylinositol 3-phosphate, a lipid that regulates membrane dynamics, protein sorting and cell signalling. Bioessays 35 (10), 900–912. 10.1002/bies.201300064. 23881848

