# Supplementary material for: Proadrenomedullin N-Terminal 20 Peptides (PAMPs) Are Agonists of the Chemokine Scavenger Receptor ACKR3/CXCR7
Source: ACS Pharmacol Transl Sci. 2021 Mar 22;4(2):813–23. doi: 10.1021/acsptsci.1c00006 (PMC8033753; doi:10.1021/acsptsci.1c00006)
Supplement: Supplementary file 1 — pt1c00006_si_001.pdf [file pt1c00006_si_001.pdf]

Supporting Information

Proadrenomedullin N-terminal 20 peptides (PAMPs) are agonists of the chemokine scavenger receptor ACKR3/CXCR7

Max Meyrath[1], Christie B. Palmer[1,2], Nathan Reynders[1,2], Alain Vanderplasschen[3], Markus Ollert[1,4], Michel Bouvier[5], Martyna Szpakowska[1,Ψ] and Andy Chevigné[1,Ψ,*]

[1] Department of Infection and Immunity, Luxembourg Institute of Health (LIH), Esch-sur-Alzette, Luxembourg

[2] Faculty of Science, Technology and Medicine, University of Luxembourg, Esch-sur-Alzette, Luxembourg

[3] Immunology-Vaccinology, FARAH, Faculty of Veterinary Medicine, University of Liège, Liège, Belgium

[4] Department of Dermatology and Allergy Center, Odense Research Center for Anaphylaxis, University of Southern Denmark, Odense, Denmark

[5] Department of Biochemistry and Molecular Medicine, Institute for Research in Immunology and Cancer (IRIC), Université de Montréal, Montreal, Canada

Ψ: these authors contributed equally to this work

* To whom correspondence should be addressed: Dr. Andy Chevigné, Immuno-Pharmacology and Interactomics, Department of Infection and Immunity, Luxembourg Institute of Health (LIH), 29, rue Henri Koch, L-4354 Esch-sur-Alzette, Luxembourg. Tel: +352 26970-336; Email: andy.chevigne@lih.lu

Table of content

Supplementary Figure 1: ACKR3 interacts specifically with all three RAMP isoforms

Supplementary Figure 2: RAMPs do not influence the responsiveness of ACKR3 and CLR to PAMP variants

Supplementary Table 1: Sequences of proADM-derived peptides and substance P and their potency in β-arrestin recruitment towards ACKR3, MrgX2 and CLR/RAMP

Supplementary Table 2: Sequences of PAMP-12-derived peptides and activity in β-arrestin-2 recruitment to ACKR3 and MrgX2

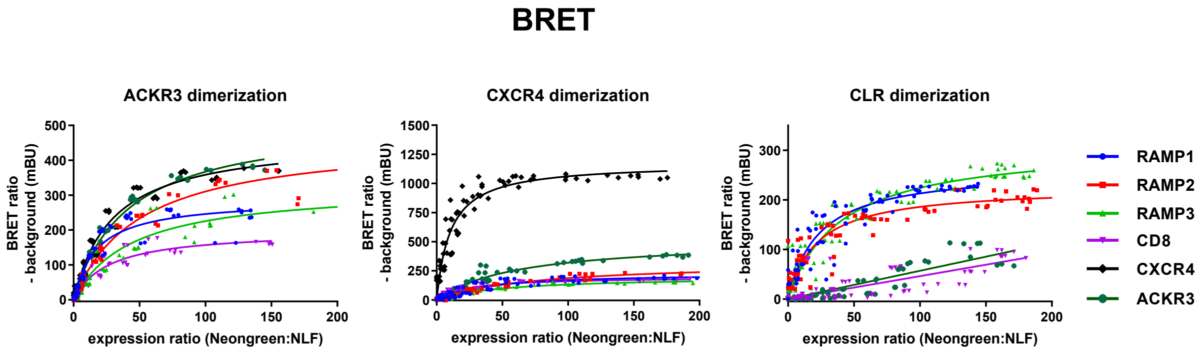

Supplementary Figure 1. ACKR3 interacts specifically with all three RAMP isoforms. BRET values measured in HEK293T cells co-transfected with a constant amount of pNLF vector encoding Nanoluciferase-tagged donor (RAMPs, CD8, CXCR4 or ACKR3) and increasing amounts of pNeonGreen vector encoding mNeonGreen-tagged acceptor (ACKR3, CXCR4 or CLR). Results represent individual measurements performed in triplicates of three independent experiments and curves were fitted with the one-site specific binding equation using GraphPad Prism.

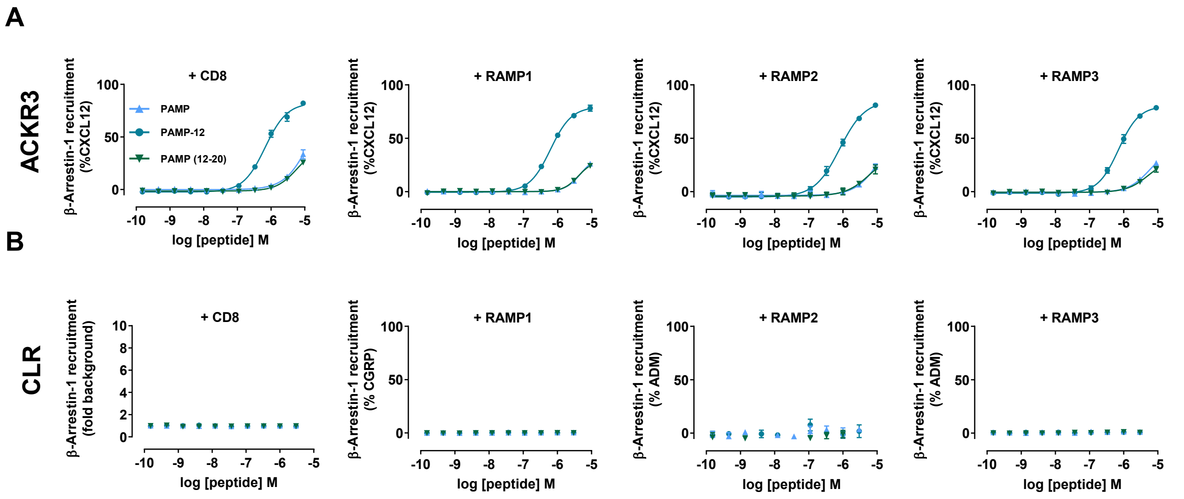

Supplementary Figure 2. RAMPs do not influence the responsiveness of ACKR3 and CLR to PAMP variants (A-B) Efficacy and potency of PAMP variants in inducing β-arrestin-1 recruitment to ACKR3 (A) and CLR (B) in HEK cells co-transfected with pcDNA3.1 encoding one of the three RAMPs or CD8 as negative control protein using NanoBiT technology. Results represent the mean ± S.E.M of three independent experiments (n = 3).

Supplementary Table 1. Sequences of proADM-derived peptides and substance P and their potency in β-arrestin recruitment towards ACKR3, MrgX2 and CLR/RAMP

Peptide	Sequence	Receptor				
		ACKR3	MrgX2	CLR/RAMP1	CLR/RAMP2	CLR/RAMP3
		pEC_{50} ±S.E.M	pEC_{50} ±S.E.M	pEC_{50} ±S.E.M	pEC_{50} ±S.E.M	pEC_{50} ±S.E.M
ADM	YRQSMNNFQGLRSFGCRFGTCTVQKLAHQIYQFTDKDKDNVAPRSKISPQGY-NH_2	approx. 5.0 - 5.3	NA	6.78 ± 0.04	7.95 ± 0.06	7.97 ± 0.06
PAMP(1-20)	ARLDVASEFRKKWNKWALSR-NH_2	< 5.0	5.21± 0.26	NA	NA	NA
PAMP(12-20)	KWNKWALSR-NH_2	< 5.0	5.80 ± 0.20	NA	NA	NA
PAMP-12	FRKKWNKWALSR-NH_2	6.08 ± 0.03	6.11± 0.05	NA	NA	NA
substance P	RPKPQQFFGLM-NH_2	NA	approx. 5.0 - 5.3	NA	NA	NA
AMY	KCNTATCATQRLANFLVHSSNNFGAILSSTNVGSNTY-NH_2	NA	NA	NA	NA	NA
IMD	TQAQLLRVGCVLGTCQVQNLSHRLWQLMGPAGRQDSAPVDPSSPHSY-NH_2	NA	NA	6.99 ± 0.04	7.32 ± 0.21	7.81± 0.08
CT	CGNLSTCMLGTYTQDFNKFHTFPQTAIGVGAP-NH_2	NA	NA	NA	NA	NA
α-CGRP	ACDTATCVTHRLAGLLSRSGGVVKNNFVPTNVGSKAF-NH_2	NA	NA	8.51± 0.06	ND	6.17 ± 0.09

NA: no activity detected in the concentration range tested
ND: not determinable

Supplementary Table 2. Sequences of PAMP-12-derived peptides and activity in β-arrestin-2 recruitment to ACKR3 and MrgX2

Sequence	Position	Receptor	
		ACKR3	MrgX2
		pEC_{50} ±S.E.M	pEC_{50} ±S.E.M
FRKKWNKWALSR	Ref	6.08 ± 0.03	6.11 ± 0.05
- RKKWNKWALSR	1	4.54 ± 0.06	5.68 ± 0.10
YRKKWNKWALSR	1	5.17 ± 0.10	6.04 ± 0.04
**A**RKKWNKWALSR	1	4.33 ± 0.23	6.03 ± 0.09
F**K**KKWNKWALSR	2	5.48 ± 0.08	5.84 ± 0.11
F**A**KKWNKWALSR	2	5.03 ± 0.08	6.00 ± 0.14
FR**R**KWNKWALSR	3	6.33 ± 0.06	5.56 ± 0.12
FR**A**KWNKWALSR	3	5.87 ± 0.06	6.01 ± 0.11
FRK**R**WNKWALSR	4	6.12 ± 0.06	5.72 ± 0.09
FRK**A**WNKWALSR	4	6.57 ± 0.06	6.18 ± 0.10
FRKK**F**NKWALSR	5	5.37 ± 0.06	6.23 ± 0.10
FRKK**Y**NKWALSR	5	5.50 ± 0.04	5.61 ± 0.09
FRKK**A**NKWALSR	5	5.12 ± 0.06	5.70 ± 0.09
FRKKW**Q**KWALSR	6	6.11 ± 0.06	5.33 ± 0.05
FRKKW**A**KWALSR	6	6.08 ± 0.06	6.32 ± 0.06
FRKKW**D**KWALSR	6	6.23 ± 0.06	6.32 ± 0.06
FRKKWN**R**WALSR	7	6.48 ± 0.06	5.71 ± 0.06
FRKKWN**A**WALSR	7	5.90 ± 0.05	6.26 ± 0.07
FRKKWNK**Y**ALSR	8	6.11 ± 0.07	6.13 ± 0.08
FRKKWNK**F**ALSR	8	5.94 ± 0.06	5.98 ± 0.06
FRKKWNK**A**ALSR	8	5.65 ± 0.07	5.60 ± 0.06
FRKKWNKW**F**LSR	9	6.29 ± 0.04	6.38 ± 0.06
FRKKWNKW**G**LSR	9	5.80 ± 0.05	5.96 ± 0.06
FRKKWNKWA**A**SR	10	5.54 ± 0.06	5.83 ± 0.05
FRKKWNKWA**F**SR	10	6.02 ± 0.07	6.13 ± 0.04
FRKKWNKWAL**A**R	11	6.04 ± 0.09	6.47 ± 0.05
FRKKWNKWAL**T**R	11	6.24 ± 0.08	6.57 ± 0.04
FRKKWNKWAL**F**R	11	6.24 ± 0.08	6.68 ± 0.07
FRKKWNKWALS**A**	12	4.95 ± 0.08	5.24 ± 0.04
FRKKWNKWALS**K**	12	5.99 ± 0.07	6.10 ± 0.06
FRKKWNKWALS -	12	5.59 ± 0.08	5.81 ± 0.05
**A**FRKKWNKWALSR	0	5.70 ± 0.05	6.05 ± 0.05
FRKKWNKWALSR-COO$^-$	C-term	5.80 ± 0.04	5.64 ± 0.04
ARLDVASEFRKKWNKWA COO$^-$- - -	PAMP1-17	3.48 ± 1.20	4.02 ± 0.40
WALSR	PAMP 16-20	4.96 ± 0.06	NA
WNKWALSR	PAMP 13-20	4.93 ± 0.06	5.69 0.04
- - - DVASEFRKKWNKWALSR	PAMP 4-20	3.75 ± 1.17	5.48 ± 0.04

Introduced mutations are in bold
Peptides used in Figure 3B-D are shown in red
NA: no activity detected in the concentration range tested
